# Othello syndrome: ¿Psychosis or dementia? A case report

**DOI:** 10.1192/j.eurpsy.2022.1676

**Published:** 2022-09-01

**Authors:** A. Franco Soler, P. Coucheiro Limeres, A. Cerame, H. Torregrosa Martínez

**Affiliations:** 1 Hospital Universitario José Germain, Psychiatry Department, Leganés, Spain; 2 Hospital Universitario Príncipe de Asturias, Neurology, Alcala de Henares, Spain

**Keywords:** Othello syndrome, CamCog, Dementia, delusional jealousy

## Abstract

**Introduction:**

Othello syndrome (OS) is a psychiatric condition consisting of delusional jealousy, and irritability. It is often associated with psychiatric or neurological disorders. The most common are delusional disorder and dementia.

**Objectives:**

The purpose of this poster is to examine the phenomenon of OS and its etiopathogenesis throughout a case report.

**Methods:**

We present the case of a 78-year-old male patient who was treated in our department due to delusional jealousy and depressive symptoms. The patient has a medical history of cardiac events in the past, being stable at the current moment. We performed a detailed psychiatric and physical history paying special attention to personality traits in the past. The patient was administered Mini Mental State Examination and CamCog (subscale of Camdex).

**Results:**

According to him and his family our patient had neither episodes of jealousy nor affective disorders. His results were: 18 in MMSE and 57 in CamCog. Both compatible with a dementia course.

**Conclusions:**

Attending our results we inferred that the OS belongs to a dementia clinical picture instead of a psychotic disorder. Therefore we decided to treat the patient with neuroleptics, with partial improvement, and to start cognitive stimulation treatment in a day centre and a short term psychological family intervention to help the family to understand and cope with the course of dementia. Thus, clinicians should keep in mind the possible organic origin of OS, especially in elderly persons, to develop an appropriate individual and familiar case approach.

**Disclosure:**

No significant relationships.

